# Limited contributions of released animals from zoos to North American conservation translocations

**DOI:** 10.1111/cobi.13160

**Published:** 2018-09-07

**Authors:** Typhenn A. Brichieri‐Colombi, Natasha A. Lloyd, Jana M. McPherson, Axel Moehrenschlager

**Affiliations:** ^1^ Centre for Conservation Research Calgary Zoological Society 1300 Zoo Road, NE Calgary Alberta T2E 7V6 Canada; ^2^ International Union for Conservation of Nature, Species Survival Commission Reintroduction Specialist Group Gland Switzerland

**Keywords:** aquaria, Central America, Caribbean, ex situ populations, reintroductions, reinforcements, zoos, acuarios, América Central y el Caribe, poblaciones ex situ, refuerzos, reintroducciones

## Abstract

With the loss of biodiversity accelerating, conservation translocations such as reintroductions are becoming an increasingly common conservation tool. Conservation translocations must source individuals for release from either wild or captive‐bred populations. We asked what proportion of North American conservation translocations rely on captive breeding and to what extent zoos and aquaria (hereafter zoos) fulfill captive breeding needs. We searched for mention of captive breeding and zoo involvement in all 1863 articles included in the North American Conservation Translocations database, which comprises journal articles and grey literature published before 2014 on conservation translocations in Canada, the United States, Mexico, the Caribbean, and Central America before 2014 as identified by a comprehensive literature review. Conservation translocations involved captive breeding for 162 (58%) of the 279 animal species translocated. Fifty‐four zoos contributed animals for release. The 40 species of animals bred for release by zoos represented only 14% of all animal species for which conservation translocations were published and only 25% of all animal species that were bred for releases occurring in North America. Zoo contributions varied by taxon, ranging from zoo‐bred animals released in 42% of amphibian conservation translocations to zero contributions for marine invertebrates. Proportional involvement of zoos in captive‐breeding programs for release has increased from 1974 to 2014 (*r* = 0.325, *p* = 0.0313) as has the proportion of translocation‐focused scientific papers coauthored by zoo professionals (from 0% in 1974 to 42% in 2013). Although zoos also contribute to conservation translocations through education, funding, and professional expertise, increasing the contribution of animals for release in responsible conservation translocation programs presents a future conservation need and opportunity. We especially encourage increased dialogue and planning between the zoo community, academic institutions, and governments to optimize the direct contribution zoos can make to wildlife conservation through conservation translocations.

## Abstract 2

*Article impact statement*: Zoos contribute animals to 14% of published North American animal conservation translocations and 25% of linked captive breeding programs.

## Abstract (es)

Contribuciones Limitadas de Animales de Zoológico Liberados en Translocaciones para la Conservación en América del Norte

**Resumen**

Conforme se acelera la pérdida de la biodiversidad, las translocaciones para la conservación ‐ como las reintroducciones ‐ cada vez son más comunes como herramientas de conservación. Las translocaciones para la conservación deben tomar individuos a liberar a partir de poblaciones criadas en cautiverio o de vida libre. Nos preguntamos cuál es la proporción de translocaciones para la conservación en América del Norte que dependen de la crianza en cautiverio y hasta qué punto los zoológicos y acuarios (de aquí en adelante, zoológicos) cumplen con las necesidades de la crianza en cautiverio. Buscamos menciones de la crianza en cautiverio y de la participación de los zoológicos en los 1863 artículos incluidos en la base de datos de Translocaciones para la Conservación de América del Norte, la cual está compuesta por artículos de revistas y literatura gris publicados sobre translocaciones en Canadá, Estados Unidos, México, América Central y el Caribe previas a 2014 identificadas por una revisión completa de la literatura. Las translocaciones para la conservación involucraron a la crianza en cautiverio en 162 (58%) de las 279 especies animales reubicadas. Cincuenta y cuatro zoológicos contribuyeron con animales para su liberación. Las 40 especies de animales criadas para luego ser liberadas por los zoológicos representaron solamente el 14% de todas las especies animales para las cuales se publicó sobre su translocación y sólo el 25% de todas las especies animales criadas para ser liberadas que existen en América del Norte. Las contribuciones de los zoológicos variaron por taxón, abarcando desde animales criados en zoológicos en el 42% de las translocaciones de anfibios hasta cero contribuciones para los invertebrados marinos. La participación proporcional de los zoológicos en los programas de crianza en cautiverio para la liberación ha incrementado desde 1974 hasta 2014 (*r* = 0.325, *p* = 0.0313), así como lo ha hecho la proporción de artículos científicos enfocados en las translocaciones co‐escritos por profesionales de los zoológicos (de 0% en 1974 a 42% en 2013). Aunque los zoológicos también contribuyen a las translocaciones para la conservación por medio de la educación, el financiamiento, y la experiencia profesional, el incremento en la contribución de animales para liberar en programas responsables de translocación para la conservación presenta una futura necesidad y oportunidad para la conservación. Alentamos de manera especial el incremento en el diálogo y en la planeación entre la comunidad de zoológicos, las instituciones académicas, y el gobierno para optimizar la contribución directa que los zoológicos pueden hacer para la conservación de la fauna por medio de las translocaciones.

## Introduction

In an effort to curb the growing loss of biodiversity, conservation translocations, “the intentional movement and release of a living organism where the primary objective is a conservation benefit” (IUCN SSC 2013), have become an increasingly important form of species management (Seddon et al. 2007; Bajomi et al. 2010; Brichieri‐Colombi & Moehrenschlager 2016; Swan et al. 2016). It is unclear how many conservation translocations are performed annually worldwide, but recent reviews indicate that over 1200 species have been subject to conservation translocations to date, based on data on North American animals (Brichieri‐Colombi & Moehrenschlager 2016), global marine taxa (Swan et al. 2016), plants (Godefroid et al. 2011; Liu et al. 2015), birds (Lincoln Park Zoo 2008; Cromarty & Alderson 2013), mammals (Van Houtan et al. 2009), amphibians (Short 2009), invertebrates and reptiles (McHalick 1999), and additional global conservation translocation databases (Soorae 2008, 2010, 2011, 2013; Armstrong et al. 2015; Soorae 2016). Conservation translocations are an important tool for addressing global conservation concerns and should be conducted responsibly when their needs and use are justified (IUCN SSC 2013).

Conservation translocations inevitably require a viable source population. Preferences are generally given to wild populations due to relatively high post‐release success in terms of survival, behavior or breeding performance across species (Letty et al. 2007). However, declines in abundance, extent of occurrence, area of occupancy or connectivity may render remaining populations too fragile to act as a continuous source (Dimond & Armstrong 2007; Todd & Lintermans 2015).

The obvious alternative to wild source populations is captive breeding. Captive breeding can be difficult due to taxon‐specific genetic, behavioral, or health challenges and postrelease success is often limited unless animals are specifically selected or adequately prepared for release (Todd & Lintermans 2015). Conversely, captive breeding can be advantageous given the ability to provide assurance against species extinction (Zippel et al. 2011), and an increased ability to target specific sex or age cohorts for releases (IUCN SSC 2013).

Institutions with long‐standing experience in captive breeding or ex situ propagation include zoos and aquaria (hereafter jointly referred to as zoos). For example, the Bronx zoo was involved in the first bison (*Bison bison*) translocation in 1907 (Kleiman 1989). Conservation‐minded breeding emerged in the 1960s (Carr & Cohen 2011) and by the 1980s transformed into the Ark paradigm, which focused on safeguarding genetic reservoirs for species or subspecies whose wild populations are under threat from human impacts (Lees & Wilcken 2009). Today, many genetically representative assurance populations are held under human care (Conde et al. 2011). However, assurance populations can only help stem the loss of biodiversity and functional ecosystems if safeguarded genes or species are ultimately returned to the wild. Accordingly, many modern zoos have in recent years increased focus on and allocated resources for threatened species recovery (Penning et al. 2009; Barongi et al. 2015), and at least two zoo associations, the World Association of Zoos and Aquariums (WAZA) and the European Associations of Zoos and Aquaria (EAZA), have formally adopted the International Union for Conservation of Nature's (IUCN) Guidelines for Reintroductions and Other Conservation Translocations (IUCN SSC 2013; Barongi et al. 2015; EAZA 2018). Species that have benefited from releases to the wild include the Arabian oryx (*Oryx leucoryx*), golden lion tamarin (*Leontopithecus rosalia*), California condor (*Gymnogyps californianus*), Kihansi spray toad (*Nectophrynoides aspergini*), Mauritius kestrel (*Falco punctatus*), Black Robin (*Petroica traversi*), and black‐footed ferret (*Mustela nigripes*), which would all be extinct without zoo intervention.

Nonetheless, it remains unclear how relevant captive breeding programs in zoos have been or could be to conservation translocations in general. A global but dated review by Beck et al. (1994) showed zoos contributed to 59% of 129 reintroduction projects involving captive bred individuals. More recently, a review based on the Global Re‐introduction Perspectives (GRP) case study series (Soorae 2008, 2010, 2011, 2013, 2016) published by the IUCN Reintroduction Specialist Group indicated that zoos were involved in only 35% of conservation translocations and contributed captive‐bred individuals for release into the wild in only 20% of cases (Gilbert et al. 2017). We wondered whether such trends are representative in general and indicative of North American activities. North America is one of the world regions with the highest conservation translocation activity globally (Seddon et al. 2014), and North American zoos accredited by the Association of Zoos and Aquaria (AZA) annually spend on average US$160 million on conservation initiatives (AZA 2018), almost half of the US$350 million raised annually for conservation by zoo and aquarium associations around the world (Conde et al. 2011; Barongi et al. 2015).

Although the GRP case studies are a valuable resource, they stem from invited submissions and are not intended as an unbiased or systematic data set of conservation translocations generally. For example, although at least 279 animals species have undergone conservation translocations in North America (Brichieri‐Colombi & Moehrenschlager 2016), GRP case studies have been published for only 48 (17%) of these. To expand understanding of the role of captive breeding and specifically of zoos in conservation translocations, we therefore mined less‐biased data gleaned from a comprehensive literature review of animal conservation translocations in North America, including Canada, the United States, Mexico, Central America, and the Caribbean (Brichieri‐Colombi & Moehrenschlager 2016). We used these data to examine what proportion of North American animal conservation translocations involve captive‐bred source populations, and of these, what percentage come from zoos. Moreover, we asked to what extent zoo professionals actively contribute to the science of conservation translocations by reporting their insights and experiences in peer‐reviewed journals. As animal‐care specialists, educators, communicators, wildlife advocates, and scientists, zoo professionals have a diversity of skills to help advance the effectiveness of conservation translocations (Barongi et al. 2015), but few zoos have traditionally seen systematic research and publication as a priority (Griffith et al. 1989; Carr & Cohen 2011).

## Methods

We used the North American Conservation Translocation (NACT) data set compiled by Brichieri‐Colombi and Moehrenschlager (2016). This data set comprises publications on North American conservation translocations involving terrestrial, marine and freshwater animals published between 1974 and December 2013. Publications were compiled using the ISI Web of Science and Academic Search Complete search engines, which primarily identified journal publications but also some grey literature, such as agency reports and newspaper and magazine articles. We searched all publications in the data set for the words “zoo*,” “*aquarium*,” “*safari*,” and “*society*.” We selected these terms by running a word frequency query on the names of all AZA accredited institutions to ensure that our search captured the majority of zoos; 85% of AZA‐accredited institutions include one or more of these terms in their name. We selected all articles that contained the terms in the author affiliation, abstract, main text, or acknowledgements sections and then recorded the species involved, type of conservation translocation, zoo name or names, type of zoo involvement (e.g., captive breeding, authorship, funding, veterinary care, etc.), source population (captive or wild), and release location. We included articles that mentioned zoo involvement but did not mention the name of the zoo, but we eliminated articles that only included a general statement about zoos (e.g., “Zoo and botanic garden managers are rethinking their organizational missions”). We used R statistical software (R Core Team 2016) to derive descriptive statistics and run Pearson's correlations on trends over time. Although we recognize that year of publication is an imperfect indicator of the timing of conservation translocations, we do not believe the variable time lag between implementation and publication introduces systematic biases in our analyses.

## Results

Our NACT data set included 1863 articles, of which 231 (12%) did not specify the source of released animals. Of the remainder, 47% (768 articles) reported animals from captive‐bred populations, 50% (816) from wild populations, and 3% (48) from both sources. Among the 279 species represented in the NACT database, 58% (162) featured in conservation translocations that released captive‐bred animals.

**Figure 1:**
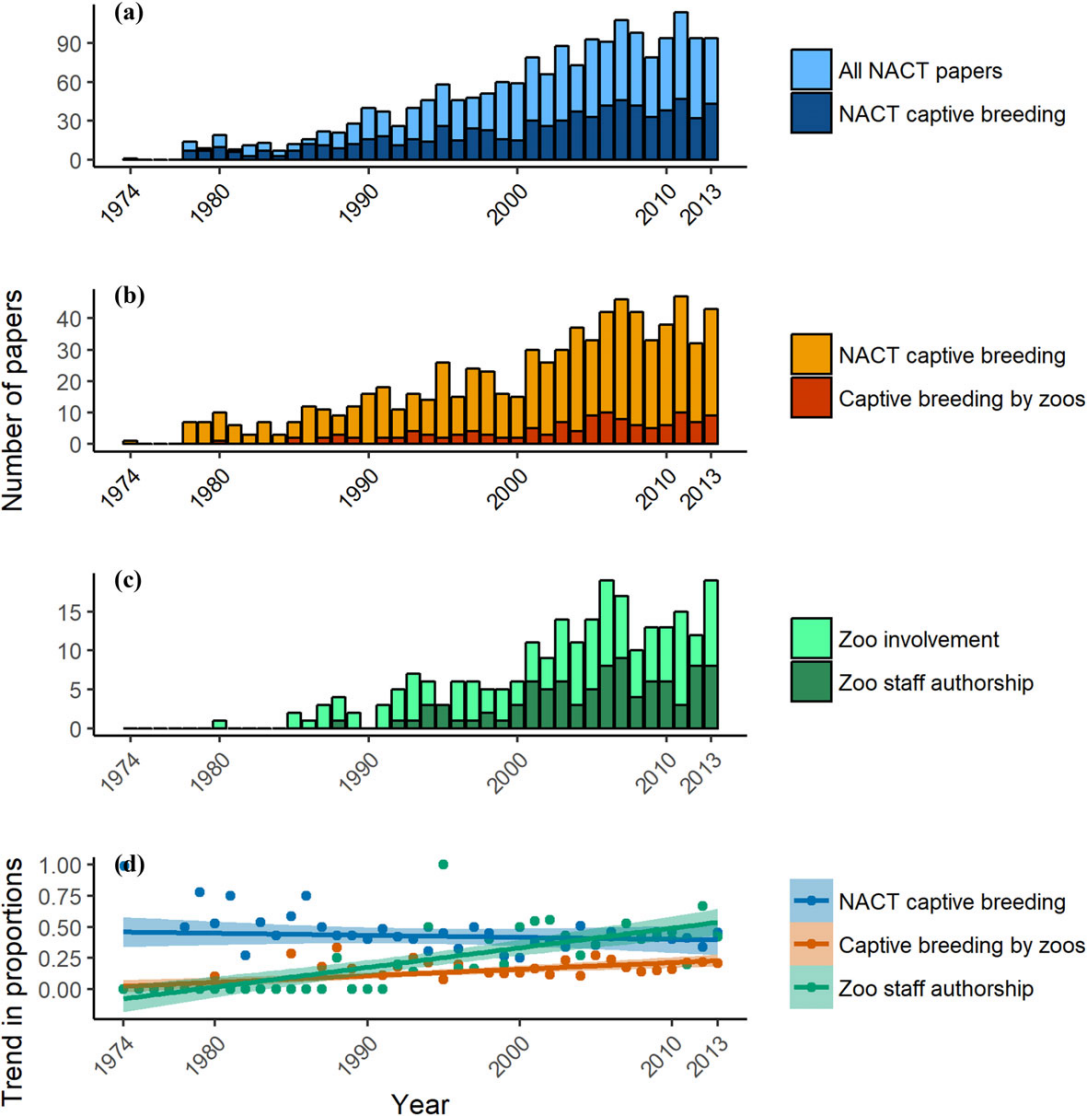
The number of papers on North American conservation translocations published annually from 1974 to 2013: (a) papers that involved captive breeding relative to all conservation translocation relevant papers in the North American Conservation Translocation (NACT) database, (b) papers that mentioned captive breeding by zoos relative to papers involving any captive breeding, (c) papers authored by zoo employees relative to papers with no authors involved with zoos, and (d) trend in proportions of NACT captive breeding, captive breeding by zoos, and authorship of researchers associated with zoos.

The proportion of species whose conservation translocations involved releases from captive‐breeding programs has not changed significantly since the 1970s (*r* = −0.137, *p* = 0.374). In fact, the proportion of publications mentioning releases from captive‐breeding, which includes multiple articles for some species, has declined slightly over time (*r* = −0.353, *p* = 0.019) (Fig. 1d) despite the increasing annual number of articles (Fig. 1a). Zoos have contributed animals toward releases of only 14% (40) of all animal species featured in published conservation translocations, and 25% of translocated species sourced from captive‐bred populations, and this proportion has not changed significantly over time (*r* = 0.229; *p* = 0.135). Of the articles that reported captive‐bred source populations, 16% (126) mentioned animals bred by zoos (Fig. 1b), and the proportion of zoo‐bred source populations increased over time (*r* = 0.325, *p* = 0.0313) (Fig. 1d). Captive breeding by zoos was more likely to contribute to North American releases for amphibians (42%), terrestrial invertebrates (29%), mammals (19%), and birds (17%) than reptiles (15%), fish (2%), or marine invertebrates (0). Of the 54 zoos involved, 50 were in North America and 4 in Europe. Of the North American Zoos, 42 were AZA accredited, representing only 18% of 230 AZA institutions.

Zoo involvement of some kind was reported in 13% (242) of all 1863 conservation‐translocation relevant papers. Zoo staff coauthored 5% of all papers in the NACT data set and 39% (94) of articles with zoo involvement. The proportion of publications with zoo staff as coauthors increased over time (*r* = 0.480, *p* < 0. 01) (Figs. 1c & d) from 0% in 1974 to 42% in 2013.

## Discussion

Our analyses provide a first insight into the significance of captive breeding for conservation translocations in North America. Over half of published North American animal conservation translocations included releases from captive‐bred populations. Yet only one‐quarter of such captive‐bred species releases come from zoos. This is lower than the 32% contribution of zoos to captive breeding for releases published in the GRP studies (Gilbert et al. 2017) and the 59% reported by Beck et al. (1994). Taxon‐specific results, in contrast, closely matched findings in Gilbert et al. (2017), with zoo‐sourced releases more likely for amphibians and terrestrial invertebrates than mammals, birds, and reptiles. The negligible proportion of zoo‐bred fish (2%) and marine invertebrate (0%) translocations identifies areas where zoos could improve their conservation contribution.

The low overall contribution by zoos to captive breeding for release may in part reflect the limited real estate available for captive breeding in zoos. Moreover, zoos individually and even jointly often hold relatively few individual animals per species (Lees & Wilcken 2009; Conway 2011) and may not house the species in greatest conservation need. Conde et al. (2011) noted that zoos housed only roughly 15% of globally threatened species, and only 6.2% of globally threatened amphibians were held in zoos in 2014 (Dawson et al. 2016). Organizations, other than zoos, that breed animals for release in greater number are often taxon‐specific facilities at governmental (e.g., Patuxant Wildlife Research Centre, Maryland), academic (e.g., University of Florida), and private organizations (e.g., San Rafael Aviaries breeding center, British Columbia). State and federal wildlife agencies in particular are heavily involved in North American conservation translocations (Beck et al. 1994; Brichieri‐Colombi & Moehrenschlager 2016; Harding et al. 2016). Zoos may be able to increase their contribution of suitable release candidates through collaborations, such as the Conservation Centres for Species Survival (C2S2 2018) and Amphibian Ark (Amphibian Ark 2017), and further study of major contributors, other than zoos, to conservation translocation is warranted to guide alternative collaboration models.

We considered only published research identified using 2 search engines, and search terms may have missed some zoos. Possibly, captive breeding by zoos focuses more on species non‐native to North America and thus makes greater contributions to translocations elsewhere. Such decisions may reflect either (or both) strategic decisions around global conservation priorities or the potentially greater public appeal of exotic species (Skibins et al. 2017). Although we suspect that releases of North American zoo‐bred animals would be less numerous on other continents than in North America, important conservation contributions have certainly been made in other regions. For example, scimitar‐horned oryx (*Oryx dammah*) (Pauling et al. 2017) were extinct in the wild, but reintroduction releases to Chad have recently been sourced in part from North American zoos. In Europe, EAZA members were involved in some capacity including funding or professional expertise in 42% of non‐European conservation translocations (Gilbert et al. 2017), but the proportion including actual animal releases is likely low. We encourage similar analyses in other regions, both those where conservation translocations are common (e.g., Oceania) and those where they are rare (e.g., South America), to help elucidate commitment to native versus non‐native species. Similarly, parallel analyses examining involvement of botanical gardens in plant conservation translocations could yield insight on patterns and approaches with potential for mutual learning opportunities.

Although zoo contributions to captive source populations used for release have grown over time (Fig. 1b), the observed trend seems too gradual to establish zoos as significant players in release‐targeted breeding any time soon. We encourage zoos to not only increase the proportion of threatened species in respective collections, but also to create network‐wide prioritization processes to identify species that would imminently benefit from conservation translocations involving captive breeding. Additionally, greater involvement likely requires an increase in the number of zoos participating in captive breeding linked to foreseeable releases. Stronger engagement should occur with government and non‐government organizations in line with the recently adopted One Plan Approach, which encourages zoo collaborations to produce holistic conservation strategies (Barongi et al. 2015). Increased engagement should not only be instigated by zoos; other institutions would likely be surprised by the untapped value zoos could bring to collaborations focused on conservation outcomes in the wild.

Of course, zoos are involved in conservation translocations in many ways other than captive breeding, such as assurance populations, research (Harding et al. 2016), and education, and overall levels of zoo involvement in our analyses matched findings by Gilbert et al. (2017). Such contributions are important and valuable to conservation translocations (Beck et al. 1994) and conservation in general. Especially promising is our finding that zoos have substantially increased their contributions to the science of conservation translocations through authorship. Increased participation in the development and documentation of scientific advances (in addition to strength in hands‐on implementation), is in line with the recent vision put forward by WAZA that “Every zoo and aquarium contributes to conservation‐relevant research to further its conservation mission, and maximises opportunities to engage in conservation‐relevant research” (Barongi et al. 2015). The government, academic, and nongovernmental organizations that commonly request, support or fund conservation translocations (Brichieri‐Colombi & Moehrenschlager 2016) are more likely to recognise and approach zoos as credible partners in conservation translocations if zoos share their existing and newly developing expertise via the peer‐reviewed scientific literature. Again, collaborations with other institutions (e.g., universities) may improve zoos’ publication output. Building on an inherent strength to make meaningful contributions to nature conservation should help zoos convincingly navigate their continued transition from (educational) entertainment parks to a genuine force for conservation.
